# The Mtr4 ratchet helix and arch domain both function to promote RNA unwinding

**DOI:** 10.1093/nar/gku1208

**Published:** 2014-11-20

**Authors:** Lacy L. Taylor, Ryan N. Jackson, Megi Rexhepaj, Alejandra Klauer King, Lindsey K. Lott, Ambro van Hoof, Sean J. Johnson

**Affiliations:** 1 Department of Chemistry and Biochemistry, Utah State University, Logan, UT 84322-0300, USA; 2 Department of Microbiology and Molecular Genetics, University of Texas Health Science Center-Houston, Houston, TX 77030, USA

## Abstract

Mtr4 is a conserved Ski2-like RNA helicase and a subunit of the TRAMP complex that activates exosome-mediated 3′-5′ turnover in nuclear RNA surveillance and processing pathways. Prominent features of the Mtr4 structure include a four-domain ring-like helicase core and a large arch domain that spans the core. The ‘ratchet helix’ is positioned to interact with RNA substrates as they move through the helicase. However, the contribution of the ratchet helix in Mtr4 activity is poorly understood. Here we show that strict conservation along the ratchet helix is particularly extensive for Ski2-like RNA helicases compared to related helicases. Mutation of residues along the ratchet helix alters *in vitro* activity in Mtr4 and TRAMP and causes slow growth phenotypes *in vivo*. We also identify a residue on the ratchet helix that influences Mtr4 affinity for polyadenylated substrates. Previous work indicated that deletion of the arch domain has minimal effect on Mtr4 unwinding activity. We now show that combining the arch deletion with ratchet helix mutations abolishes helicase activity and produces a lethal *in vivo* phenotype. These studies demonstrate that the ratchet helix modulates helicase activity and suggest that the arch domain plays a previously unrecognized role in unwinding substrates.

## INTRODUCTION

To maintain correct gene expression in the cell, the integrity of RNA must be tightly regulated through RNA processing, turnover and surveillance pathways (1–3). Several disease states are linked to defects in RNA quality control mechanisms, including neurodegenerative diseases, congenital diseases and cancer (4–8). The eukaryotic exosome, which contains both endonuclease and 3′-5′’ exonuclease activities, plays a critical role in a wide variety of RNA processing and degradation pathways (9–14). Regulation of this activity involves multiple protein cofactors including the Ski2-like RNA helicases Mtr4 (nucleus) and Ski2 (cytosol). Mtr4 acts as a subunit of the TRAMP complex to activate exosomal degradation of several pre-mRNAs, and ncRNAs such as aberrant, antisense, CUTs (cryptic unstable transcripts), and intronic RNAs (reviewed in (12,15–20)). TRAMP, which is composed of a poly(A) polymerase (Trf4 or Trf5), a zinc knuckle RNA-binding protein (Air2 or Air1) and Mtr4, promotes 3′-5′ exosomal degradation by adding a short 4-5 nucleotide poly(A) tail to the 3′ end of RNA (21–25). Mtr4 also acts independent of other TRAMP subunits to facilitate 3′ end maturation of 5.8S rRNA by the exosome (21). The helicase activity of Mtr4 is proposed to resolve secondary structures and remove proteins associated with the RNA, thus facilitating delivery of single-stranded RNA (ssRNA) to the exosome (15,17,26).

Kinetic studies indicate that Mtr4 has a higher affinity for RNA substrates containing a polyadenylated 3′ end relative to non-poly(A) sequences. Additionally, binding characteristics differ between the two types of RNA substrates when in the presence of nucleotides, suggesting that Mtr4 interacts with poly(A) sequences using a mechanism distinct from that employed to bind non(A) sequences (27,28). Notably, both Mtr4 and TRAMP show an unwinding preference for substrates with a 3′ overhang containing a poly(A) tail (29). Maximal binding affinity and unwinding activity is observed when the poly(A) tail length is approximately 5 nucleotides in length (27,29). Furthermore, the polyadenylation activity of TRAMP is restricted by Mtr4 to maintain this optimal tail length in targeted RNAs (24). This *in vitro* observation is consistent with a UV cross-linking study in yeast, which determined that Trf4 substrates contain an average poly(A) tail length of 5 nucleotides, supporting the conclusion that poly(A) tail length is regulated *in vivo* (25). These studies suggest Mtr4 contains a fine-tuned mechanism that senses the number and identity of 3′ end poly(A) tracts through a distinct binding mode, which modulates the polymerase and unwinding activities of TRAMP. It is unclear however, how Mtr4 senses the length and identity of the sequence, and how this sensing is coupled to unwinding.

Recent crystal structures of Mtr4, including apo and RNA-bound forms, and several related Ski2-like and DEAH/RHA-box helicase structures provide insight into the general features employed by these helicases to bind and translocate along nucleic acid substrates (30–39). Although each helicase exhibits unique features and accessory domains, they all contain a common core structure composed of two RecA domains (domains 1 and 2 in Mtr4), a winged helix domain (domain 3 in Mtr4) and a 7-8 helix bundle domain (ratchet domain or domain 4 in Mtr4) (40). The RecA domains contain conserved sequence motifs that bind nucleic acid, and bind and hydrolyze ATP (41). The Ski2-like Hel308 DNA helicase structure (31) indicates that a β-hairpin loop within the second RecA domain facilitates strand separation as the nucleic acid enters the helicase core. The 3′ single-stranded nucleic acid then traverses the RecA domains and interacts with domain 4 before exiting the helicase at the base. Multiple interactions are observed within domain 4, particularly along the ratchet helix where nucleotides stack with W599 and R592 in a manner that is thought to facilitate DNA translocation in Hel308 (31). Not surprisingly, deletion of domain 4 abolishes helicase activity in Hel308 (31), and the analogous mutant in Mtr4 is inviable *in vivo* (42). In a related Ski2-like helicase Brr2 a R1107A point mutation in domain 4 (equivalent to position W599 in Hel308) conferred a slow growth phenotype and loss of *in vitro* activity (42,43). Mutations along the Brr2 ratchet helix are also associated with autosomal dominant retinitis pigmentosa (adRP) (44). Point mutations in domain 4 of Mtr4 and other Ski2-like helicases display slow growth phenotypes and loss of *in vitro* unwinding activity (43,45–47). Although domain 4 appears to play an important role in helicase activity, a mechanistic description of domain 4 function is lacking, particularly for Ski2-like RNA helicases.

In an effort to better define Mtr4-RNA interactions, we have investigated amino acid residues along the ratchet helix of domain 4. Sequence and structural analysis reveals discrete conservation patterns in Mtr4, Ski2-like and DEAH/RHA-box helicases. Mutagenesis studies demonstrate that R1030 and E1033 play important, but distinct roles in sequence recognition and helicase activity on poly(A) and non(A) substrates. *In vivo* analysis further underscores the importance of ratchet helix residues for cellular function. Additionally, we demonstrate arch domain involvement in unwinding activity when combined with either ratchet helix point mutation, suggesting a mechanism for RNA substrate recognition and unwinding by Mtr4 that involves both the ratchet helix and arch domain.

## MATERIALS AND METHODS

### Structural analysis and conservation scoring of Ski2-like and DEAH/RHA-box helicases

**Figure 1.**
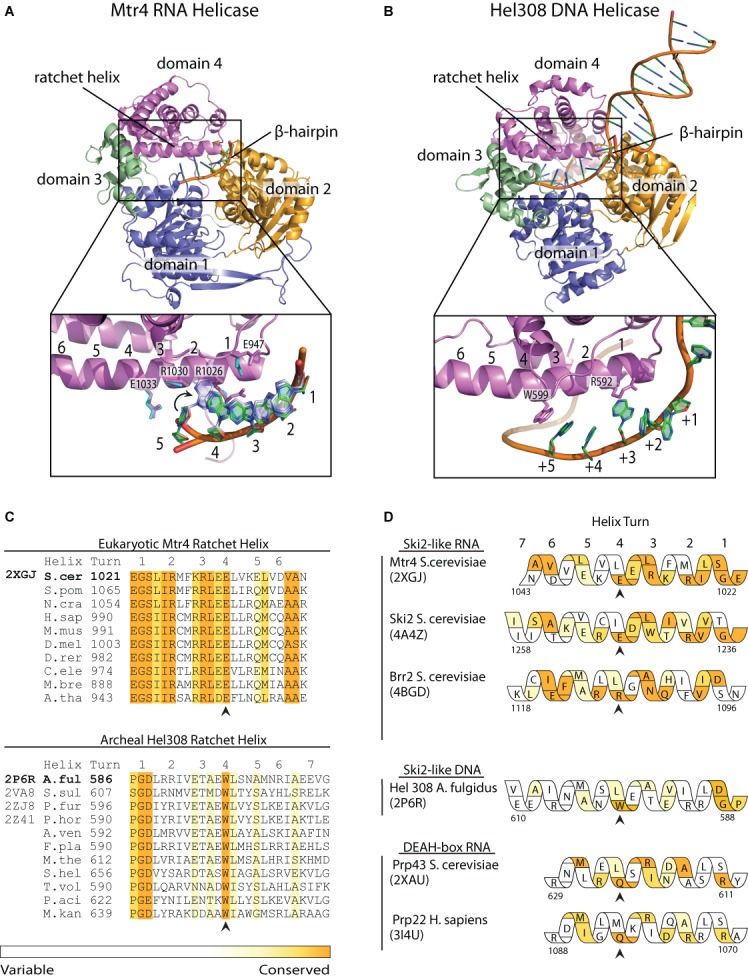
Conserved ratchet helix residues interact with nucleic acid. (**A**) The RNA-bound Mtr4 structure (PDB 2XGJ) is colored by domain. (Inset) Helix bundle domain (domain 4 or ratchet domain) residues that interact with poly(A) RNA are shown as sticks. For reference, the ratchet helix is numbered by helical turn in an N- to C-terminal direction. Aligned residues (cyan) and RNA bases (light blue) from a second molecule in the asymmetric unit are also indicated. Nucleotides are labeled 1 through 5 in a 5′-3′ direction. (**B**) The DNA-bound Hel308 structure (PDB 2P6R) is colored as in (A). The inset image highlights the pi stacking interactions of ratchet helix residues W599 and R592 shown as sticks with bases of ssDNA. Nucleotides downstream of the strand separation β-hairpin are labeled +1 through +5. (**C**) Alignment and conservation scores (calculated using Consurf (50)) of eukaryotic Mtr4 and archaeal Hel308 ratchet helix sequences. Conservation is colored strictly conserved as orange, to variable as white. Extensive conservation at helical turn 4 is highlighted with an arrow. Alignment of Mtr4 includes 10 model eukaryotic species (S.cer, *Saccharomyces cerevisiae*; S.pom, *Schizosaccharomyces pombe*; N.cra, *Neurospora crassa*; H.sap, *Homo sapiens*; M.mus, *Mus musculus*; D.rer, *Danio rerio*; D.mel, *Drosophila melanogaster*; C.ele, *Caenorhabditis elegans*; M.bre, *Monosiga brevicollis*; A.tha, *Arabidopsis thaliana*). Alignment of Hel308 includes the sequences of crystal structure homologs and seven other archeal sequences (A.ful, *Archaeoglobus fulgidus*; S.sul, *Sulfolobus solfataricus*; P.fur, *Pyrococcus furiosus*; P.hor, Pyrococcushorikoshi; A.ven, *Archaeoglobus fulgidus*; F. pla, *Ferroglobus placidus*; M.the, *Methanosaeta thermophila* S.hel, Staphylothermus hellenicus; T.vol, *Thermoplasma volcanium* P.aci, *Candidatus Parvarchaeum acidophilus*; M.kan, *Methanopyrus kandleri*). (**D**) Conservation of each residue along the ratchet helix of Ski2-like and DEAH/RHA-box helicases is depicted with Consurf scores colored as in (C). Complete sequence alignments are shown in Supplementary Figure S2. Conservation at helical turn 4 is highlighted with an arrow. Structures in (A) and (B) were rendered using PyMOL (62).

The helix bundle domain (domain 4) of archaeal Hel308 (PDB: 2P6R) (31) was used as bait in a DALI search (48) to find structures containing a helix bundle domain with an associated ratchet helix. Conservation of eukaryotic helicases was determined by multiple sequence alignment of model organisms in CLUSTALW (Figure 1C) (49) and conservation scoring with the ConSurf server (50). For the archaeal Ski2-like DNA helicase Hel308, 98 archaeal sequences including the sequences of existing Hel308 structures were retrieved and scored using the ConSurf server (50). ConSurf output scores of 7-9 (on a scale of 1-9) were considered conserved and are represented as colored regions in Figure 1C and D. Molecular graphics were rendered using PyMOL (62).

### Mutagenesis, protein expression and protein purification

Point mutations of Mtr4 wild-type (Mtr4^WT^) were made using a modified version of the QuikChange (Agilent) site-directed mutagenesis procedure (51). The expression and purification of Mtr4^WT^ and mutant Mtr4 proteins was carried out as performed previously (30). While most of the Mtr4 mutants purified essentially as wild-type, the Mtr4^R1030A-archless^ mutant exhibited poor solubility yielding much lower amounts of protein. Protein concentration was determined using a NanoDrop spectrophotometer (Thermo Fisher) with an extinction coefficient for Mtr4 of 89 450 M^−1^ cm^−1^ (calculated using ExPASy ProtParam (52)). Expression and purification of TRAMP complexes were performed essentially as described by Jia *et al*. (24). Full-length Mtr4^WT^ and mutants were cloned into a pET151/D-TOPO vector. A pETDuet-1 vector containing full-length Air2 and full-length Trf4 (with an active site knockout mutation of D236A/D238A) was obtained from the Jankowsky lab. All proteins were recombinantly expressed in an *Escherichia coli* BL21-codon+-(DE3)-RIL cell line (Stratagene). Cell lysis was performed by lysozyme treatment and sonication of frozen cell pellets. Cell lysis and clarification were performed separately for Mtr4^WT^ and Trf4/Air2, at which point the soluble fractions were combined. Cobalt affinity, FLAG affinity and NAP-25 gel filtration was used to purify TRAMP complexes at 4°C. Final purification buffer consisted of 50 mM sodium phosphate (pH 7.0), 10% glycerol, 200 mM sodium chloride and 10 μM zinc chloride.

### RNA substrate design and purification

RNA substrates were designed to mimic the unwinding substrates used by Jia *et al*. (29). Two 22 nucleotide ssRNAs (bottom strand, each with a unique 3′ end) were incubated independently with a complementary 16 nucleotide ssRNA (top strand) at 95°C for 10 min after which samples were slowly annealed to room temperature.

All RNAs used in this study were purchased from Integrated DNA Technologies (IDT). The substrate sequences are as follows with duplex regions underlined: R16 (top strand of all three substrates), 5′-AGCACCGUAAAGACGC-3′; R22A (poly(A) overhang), 5′-GCGUCUUUACGGUGCUUAAAAA-3′; R22R (non(A) overhang), 5′-GCGUCUUUACGGUGCUUGCCUG-3′. The 16 nucleotide top strand was radiolabeled using γ-^32^P ATP and T4 polynucleotide kinase and quenched by heating to 95°C before annealing. The RNA substrates were purified by native polyacrylamide gel electrophoresis, gel extraction and ethanol precipitation. Annealed substrates with the R22A bottom strand are referred to as a poly(A) substrate, while the annealed substrates with the R22R bottom strand are referred to as a non(A) substrate.

### Unwinding assay

Pre-steady state unwinding assays were performed essentially as described (29). A radiolabeled 16 nucleotide top strand was displaced over time when incubated with Mtr4^WT^ and saturating levels of ATP. Reactions were carried out at 30°C in a controlled water bath. The buffer used was 40 mM MOPS (pH 6.5), 100 mM NaCl, 0.5 mM magnesium chloride, 5% glycerol, 0.01% nonidet-P40 substitute (Amresco), 2 mM dithiothreitol and 1 U/μl of Ribolock (Thermo Fisher). Reactions were allowed to incubate for 5 min with ∼0.2 nM RNA (final concentration) and the indicated concentration of Mtr4^WT^ or Mtr4 mutant protein. Reactions were initiated by the addition of ATP and MgCl_2_ at saturating concentrations (1.6 mM each; the *K*_m_ for ATP is 0.39 mM (28)). At specified time points, aliquots of the reaction were removed and quenched at a 1:1 ratio with buffer containing 1% sodium dodecyl sulfate, 5 mM ethylenediaminetetraacetate (EDTA), 20% glycerol, 0.1% bromophenol blue and 0.1% xylene cyanol. Aliquots were run on a native 15% polyacrylamide TBE gel at 100 V for 115 min. Radioactivity was visualized as performed previously (30). Gels were wrapped in cellophane and exposed to X-ray film or phosphor screen. Film was developed and then quantified using multigauge software; phosphor screen was developed by a Storm Phosphorimager (Amersham Biosciences) and quantified using ImageQuant software. Calculations of the observed rate constants (*k*_unw_), and amplitudes (*A*) were performed using an integrated first-order rate law. Curve fits were made to data collected in triplicate, as employed previously (Fraction unwound = *A*(1−exp(-*k*_unw*_t)))(29,30,53). The *k*_unw_^max^ and *K*_1/2_ values were calculated using best fit curves (29), with the equation, *k*_unw_ = *k*_unw_^max^,_E_ [E]/([E] + *K*_1/2_,_E_); where [E] is enzyme concentration, *K*_1/2_,_E_ is functional affinity (29) and *k*_unw_^max^,_E_ is the unwinding rate constant at enzyme saturation.

### Binding assay

Binding analysis of Mtr4 to RNA was carried out using fluorescence anisotropy. A fluorescein label was added to the 3′ end of the R16 RNA, and was annealed to the longer R22A substrate containing a 3′ poly(A) overhang. Binding reactions were buffered in 40 mM MOPS (pH 6.5), 100 mM sodium chloride, 0.5 mM magnesium chloride, 5% glycerol, 0.01% nonidet-P40 substitute and 2 mM dithiothreitol. Concentration of the fluorescently labeled duplexed RNA was held constant at 60 nM with increasing concentrations of protein (varied depending on the binding affinity of each mutant). Mtr4^WT^ and mutants were incubated with RNA substrate for 1–5 min to reach equilibrium before each measurement was taken. At all protein concentrations tested, the protein was in excess over RNA and the RNA was <10% of the *K*_d_ to allow for fitting of the data using a ligand binding, one-site saturation equation (*F* = [L]/(*K*_d_ + [L]), where [L] is the concentration of Mtr4). Anisotropy was measured on a Synergy H4 Hybrid Multi-Mode Microplate Reader (BioTek) with an excitation at 485 nm and an emission at 528 nm at 30°C. Sigmaplot (Systat Software) was used for curve analysis and *K*_d_ determination.

### ATPase assay

Mtr4 ATPase activity was measured using a malachite green assay, adapted from previously published protocols (28,54). Absorbance was monitored at 650 nm with a VERSA max tunable plate reader (Molecular Devices). An increase in absorbance at 650 nm correlates to free inorganic phosphate and corresponds to ATP hydrolysis. Each reaction contained 25 mM Tris (pH 7.5), 10 mM magnesium acetate, 2 mM β-mercaptoethanol, 0.3 μM of protein and 0.6 μM of RNA. The reactions were initiated with the addition of saturating concentrations of ATP and MgCl_2_ (1.33 mM each) with time points taken at 0, 5, 10, 15 and 20 min. A 5X quenching solution (250 mM EDTA) was then mixed with each sample to reach a final concentration of 50 mM EDTA. The 2X malachite green solution (650 μM malachite green oxalate and 10 μM sodium molybdate) was added in a 9:1 excess to the sample and incubated for 17 min to complete the reaction before reading the absorbance at 650 nm. Initial rates ([Pi] μM min^−1^) were calculated by fitting a linear trend line to the absorbance values at the different time points using KaleidaGraph (Synergy Software). To determine the ATPase activity enhancement due to RNA with Mtr4^WT^and Mtr4 mutants, reactions without RNA were used to obtain background values.

### Yeast plasmids

The plasmids for expression of Mtr4^WT^or Mtr4 ratchet helix mutants contained the same upstream promoter and downstream sequence as used previously (30). The same Mtr4^WT^ expression plasmids pAv673 (a URA3 CEN plasmid; (55)), and pAv675 (a LEU2 CEN plasmid; (55)) were used as in (30). Plasmids expressing ratchet helix mutants are simply point mutants of pAv675.

### Yeast growth assays

An Mtr4 deletion strain of *Saccharomyces cerevisiae* complemented with an Mtr4^WT^ copy plasmid containing a URA3 selectable marker was transformed with Mtr4^WT^ or Mtr4 ratchet helix mutant plasmids. Transformants were grown in Synthetic Complete-LEU (SC-LEU) liquid media overnight at 30°C to allow for random loss of the URA3 plasmid. Liquid cultures were serially diluted 5-fold and spotted onto control plates (SC-LEU) or 5-fluoro-orotic acid (5-FOA; to select cells that had lost the Mtr4^WT^ plasmid containing the URA3 marker) and grown at 20, 30 or 37°C.

### Crystallographic refinement

During our comparison of the 3.4 Å apo Mtr4 structure (PDB: 3L9O) and the 2.9 Å RNA-bound structure (PDB: 2XGJ), it became apparent that improvements in sequence register and connectivity could be made in some regions of the lower resolution structure. Remodeled regions were primarily confined to the N-terminus, the fist (or KOW) region of the arch domain and a few loops (Supplementary Figure S1). The electron density maps resulting from the improved model revealed additional electron density for several side chains, including several residues along the ratchet helix, which were then incorporated into the model. Model building was performed using Coot (56). PHENIX was used to perform individual B-factor, positional and TLS refinement (57). Secondary structure restraints were used throughout refinement. This process achieved an ∼5% improvement in *R*/*R*_free_ from the original Mtr4 structure (PDB: 3L9O), with final values of 0.248/0.299 (Supplementary Figure S1). The revised coordinates have been deposited at the Protein Data Bank under accession code 4QU4, and are linked to the original submission (3L9O).

## RESULTS

### Structural analysis of the Mtr4 RNA-binding path reveals distinct modes of substrate binding

In the RNA-bound structure of Mtr4, two molecules are observed in the asymmetric unit (38). In both molecules, a 5 nucleotide poly(A) RNA substrate interacts with the canonical helicase motifs of the RecA domains 1 and 2 through multiple phosphate backbone interactions, similar to that observed in Hel308 (Supplementary Figure S2) (31). Domain 4 is positioned opposite domains 1 and 2, and interacts directly with the RNA bases. The primary base interactions in Mtr4 are with E947 (located on a loop above the ratchet helix) R1026, R1030 and E1033, occupying one face of the ratchet helix. Notably, each of these base interactions appears to be mediated through hydrogen bonds, whereas interactions in Hel308 generally involve base stacking (Supplementary Figure S2). The direct protein-nucleotide base interactions observed in the Mtr4 crystal structure suggest that the function of the ratchet helix may not be restricted to RNA translocation (by analogy to Hel308), but may also involve RNA sequence recognition.

### Ratchet helix residues are conserved in Ski2-like/DExH-box helicases

We next examined the conservation of the residues along the ratchet helix for the Ski2-like Mtr4, Ski2, Brr2 and Hel308 helicases, and the DEAH/RHA-box Prp22 and Prp43 helicases (36,37,43,46). CLUSTALW was used to align a diverse set of eukaryotic sequences for each helicase (archaeal sequences were used for Hel308) (Figure 1C and Supplementary Figure S3) (49). Conservation scores were calculated using the ConSurf server (50). Extensive conservation is observed along the entire ratchet helix for the Ski2-like RNA helicases (Mtr4, Ski2 and Brr2) (Figure 1C and D). Less conservation is observed for Hel308 and the DEAH/RHA-box RNA helicases Prp22 and Prp43. In the case of Hel308, position W599 is the only strictly conserved ratchet helix residue observed to interact with nucleic acid. The conserved residues observed at the N-termini of each helix are involved in interactions with domain 2 and generally do not interact directly with nucleic acid.

Although no residue along the ratchet helix is universally conserved throughout Ski2 and DEAH/RHA-box helicases, conservation patterns are clearly evident. The most striking feature is that the fourth turn of the ratchet helix (counting from the N-terminus) is strictly conserved in a helicase-specific manner (Figure 1D). Mtr4 and Ski2 always have a glutamate at the same position on the fourth turn (E1033 in Mtr4; E1247 in Ski2), Brr2 has an arginine (R1107), Prp22 and Prp43 have a glutamine (Q1081 in Prp22; Q622 in Prp43), and Hel308 has a tryptophan (W599). Among the Ski2-like RNA helicases, we note similar conservation patterns at the second and third turns of the ratchet helix. In the case of Mtr4, both positions are always arginines (R1026 and R1030).

### R1030 and E1033 play distinct roles in unwinding

The interaction of R1030 and E1033 with RNA observed in the Mtr4 structures combined with the strong conservation at each of these positions in Ski2-like RNA helicases suggested that these residues might be important for Mtr4 activity. To assess the role of these residues in Mtr4 function, we mutated each position in *S. cerevisiae* Mtr4 to alanine (R1030A, E1033A). E1033 was also mutated to tryptophan (E1033W) to mimic the sequence observed in Hel308. Pre-steady state unwinding assays and calculations were performed using a helicase assay developed previously to characterize the unwinding activity of Mtr4 and other helicases (29,58). The assay detects the displacement of a ^32^P labeled top strand from a complementary bottom strand with a 3′ single-stranded extension of six nucleotides (Supplementary Figure S4).

**Figure 2.**
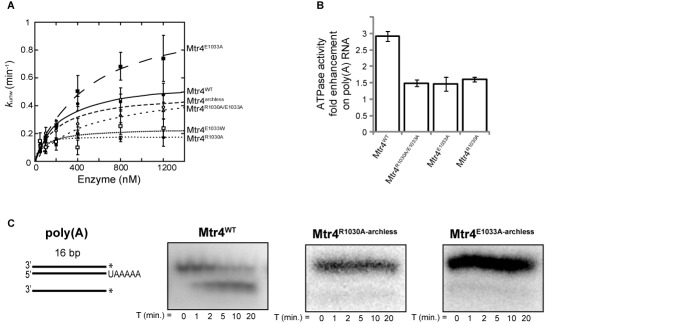
Effect of ratchet helix mutations on Mtr4 activity. (**A**) Poly(A) RNA unwinding rate (*k*_unw_) constants determined for Mtr4^WT^ and Mtr4 mutants, plotted as a function of Mtr4 concentration. Best fit curves to the data were calculated as described in ‘Materials and Methods’. Data presented here represent averages from three independent experiments; error bars represent SD. (**B**) Enhancement of ATPase activity in the presence of poly(A) RNA for Mtr4^WT^ and ratchet helix mutants. Data presented here represent averages from two independent experiments; error bars represent SD. (**C**) RNA unwinding gels of a poly(A) tail for Mtr4^WT^, Mtr4^R1030A-archless^ and Mtr4^E1033A-archless^ reveal no unwinding activity for the Mtr4^R1030A-archless^ and Mtr4^E1033A-archless^ double mutants.

Using a 3′ polyadenylated substrate (poly(A)), we observed a smaller unwinding constant (*k*_unw_) for the Mtr4^R1030A^ mutant at 800 nM protein than that observed for wild-type enzyme (Supplementary Figure S4). In contrast, the Mtr4^E1033A^ protein demonstrated a higher *k*_unw_ at 800 nM than Mtr4^WT^ (Supplementary Figure S4). Mutation of E1033 to a tryptophan significantly impaired unwinding of a poly(A) substrate (Figure 2A and Table 1). Unwinding rate constants (*k*_unw_) at several enzyme concentrations were determined for the ratchet helix mutants to obtain the strand-separation rate constants at enzyme saturation (*k*_unw_^max^) (Figure 2A and Table 1). Compared to wild-type, the Mtr4^R1030A^ and Mtr4^E1033W^ mutants displayed a lower *k*_unw_^max^ and the Mtr4^E1033A^ mutant displayed a higher *k*_unw_^max^, demonstrating that residue identity at specific ratchet helix positions directly influences the strand-separation rate constant. No significant differences were observed in functional affinities (*K*_1/2_) between a poly(A) and a non(A) substrate for each individual mutant within the error reported. Additionally, all ratchet helix mutants displayed lower RNA enhanced ATPase activity than Mtr4^WT^, regardless of whether their unwinding rate constant was faster or slower (Figure 2B). This result suggests that at the current level of ATPase activity, ATPase and unwinding activity are minimally correlated. A similar lack of correlation between ATPase and unwinding has been observed in mutants of the NS3 helicase (59). A more dramatic reduction in ATPase activity, however, is expected to affect unwinding activity. Mutation of residues D262 and E263 to alanine, which are directly involved in ATP hydrolysis (Mtr4^D262A/E263A^), completely abolishes ATPase activity and unwinding activity (data not shown).

**Table 1.** Kinetic parameters (*k*_unw_^max^ and *K*_1/2_) of Mtr4 mutants for unwinding poly(A) and non(A) substrates^a^

	poly(A)	non(A)	poly(A)	non(A)
Enzyme	*k*_unw_^max^ (min^−1^)	*k*_unw_^max^ (min^−1^)	*K*$\frac{1}{2}$ (nM)	*K*$\frac{1}{2}$ (nM)
Mtr4^WT^	0.59 ± 0.05	0.34 ± 0.05	252 ± 60	255 ± 116
Mtr4^archless^	0.49 ± 0.07	0.34 ± 0.08	221 ± 93	260 ± 164
Mtr4^R1030A^	0.18 ± 0.02	0.16 ± 0.03	51 ± 26	129 ± 92
Mtr4^E1033A^	1.08 ± 0.09^b^	1.17 ± 0.24^b^	504 ± 93	1415 ± 468
Mtr4^E1033W^	0.22 ± 0.07	undetermined	484 ± 360	undetermined
Mtr4^R1030A/E1033A^	0.52 ± 0.1	0.41 ± 0.04	498 ± 216	269 ± 81
Mtr4^R1030A-archless^	n.d.	n.d.	n.d.	n.d.
Mtr4^E1033A-archless^	n.d.	n.d.	n.d.	n.d.

Data presented here represent averages from three independent experiments; error bars represent SD. n.d., no unwinding activity detected.

^a^Methodologies and equations used to derive kinetic constants are found in the ‘Materials and Methods’ section.

**Figure 3.**
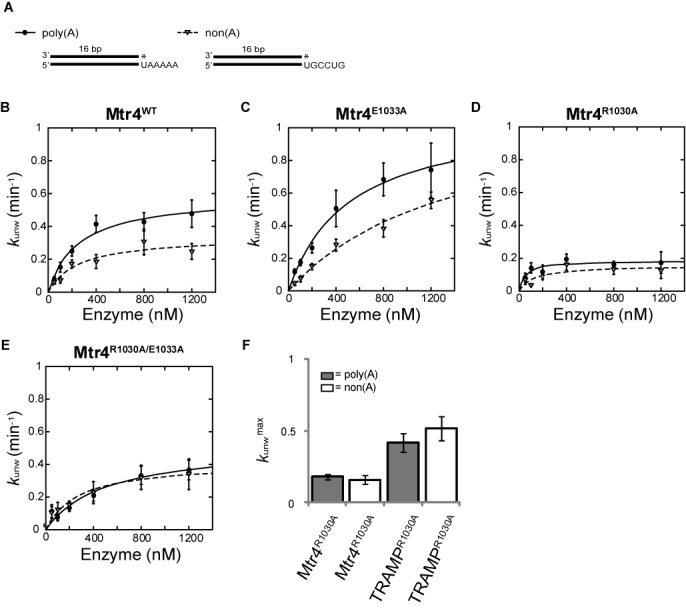
Role for R1030 in 3′ poly(A) sequence recognition. (**A**) Comparison of the poly(A) and non(A) RNA substrates used in this study. Each substrate is composed of identical 16 bp duplex regions and variable 3′ end overhangs (see ‘Materials and Methods’ for full sequence details). (**B**) The Mtr4^R1030A^ mutant loses the ability to discriminate between a poly(A) and non(A) RNA substrate. Unwinding rate (*k*_unw_) constants determined for Mtr4^WT^ (A) and Mtr4 mutants (B, **C**, **D**, **E**), plotted as a function of Mtr4 concentration using poly(A) (solid line) and non(A) (dashed line) RNA substrates. Although Mtr4R^1030A/E1033A^ restores unwinding activity to wild-type levels, no difference is observed between poly(A) and non(A) substrates (E). Best fit curves to the data were calculated as described in ‘Materials and Methods’. Data presented here represent averages from three independent experiments; error bars represent SD. (**F**) Comparison of *k*_unw_^max^ values for Mtr4 and TRAMP complexes containing the R1030A mutation (Mtr4^R1030A^ and TRAMP^R1030A^) shows that TRAMP^R1030A^ also loses the ability to discriminate between poly(A) and non(A) RNA substrates. Data presented here represent averages from four independent experiments; error bars represent SD.

^b^Since the curve for the Mtr4^E1033A^ catalyzed unwinding never reached saturation, calculated *k*_unw_^max^ rates between poly(A) and non(A) appear within error, although a clear distinction between RNA substrates is observed at every tested concentration (see Figure 3).

Since *in vivo* effects of the Mtr4 mutants may be expected to arise through interactions in the TRAMP complex, we examined the Mtr4^R1030A^ mutant in the context of TRAMP. Trf4 and Air2 have been previously shown to stimulate the unwinding rate of Mtr4^WT^ on poly(A) and non(A) substrates (29). We observe a similar Trf4-Air2 dependent stimulation of unwinding activity with the Mtr4^R1030A^ mutant (Table 2). We also observe the same relative effects on unwinding rate as observed with Mtr4 alone; TRAMP^R1030A^ is ∼2.5 fold slower than TRAMP^WT^.

**Table 2.** Kinetic parameters (*k*_unw_^max^ and *K*_1/2_) of TRAMP^WT^ and TRAMP^R1030A^ for unwinding poly(A) and non(A) substrates^a^

	poly(A)	non(A)	poly(A)	non(A)
Enzyme	*k*_unw_^max^ (min^−1^)	*k*_unw_^max^ (min^−1^)	*K*$\frac{1}{2}$ (nM)	*K*$\frac{1}{2}$ (nM)
TRAMP^WT^	1.96 ± 0.75	0.57 ± 0.41	497 ± 268	238 ± 300
TRAMP^R1030A^	0.42 ± 0.06	0.52 ± 0.08	24 ± 19	32 ± 23

Data presented here represent averages from at least three independent experiments; error bars represent SD. The non-catalytically functional Trf4(D236/D238A) mutant was used for all TRAMP preps.

^a^Methodologies and equations used to derive kinetic constants are found in the ‘Materials and Methods’ section.

### R1030 is involved in discrimination between poly(A) and non(A) sequences

To study the effects of different RNA sequences on the unwinding activity of Mtr4 ratchet helix mutants, we determined unwinding rate constants for a non-polyadenylated substrate (non(A)) used recently to characterize Mtr4^WT^ sequence preferences (29). Mtr4^WT^ and Mtr4^E1033A^ enzymes showed an unwinding preference in *k*_unw_^max^ for the poly(A) substrate over the non(A) substrate at all enzyme concentrations tested (Figure 3B and C). In contrast, the Mtr4^R1030A^ mutant displayed roughly identical *k*_unw_ values for the poly(A) and non(A) substrates at each concentration (Figure 3D and Table 1). To further characterize the impact of the E1033 and R1030 mutation, we tested the double alanine mutant Mtr4^R1030A/E1033A^ for unwinding activity. The Mtr4^R1030A/E1033A^ mutant unwound the substrate faster than Mtr4^R1030A^ alone; however, it did not regain the ability to differentiate between a poly(A) and a non(A) substrate (Figure 3E and Table 1). Additionally, when Trf4 and Air2 are combined with Mtr4^R1030A^, the resulting TRAMP^R1030A^ complex exhibits an increased unwinding rate but is unable to differentiate between a poly(A) and non(A) substrate (Figure 3F and Table 2). Thus, R1030 plays a role in discriminating between the poly(A) and non(A) sequences, both in Mtr4 alone and in a TRAMP context.

### R1030 and E1033 are important for Mtr4 function *in vivo*

**Figure 4.**
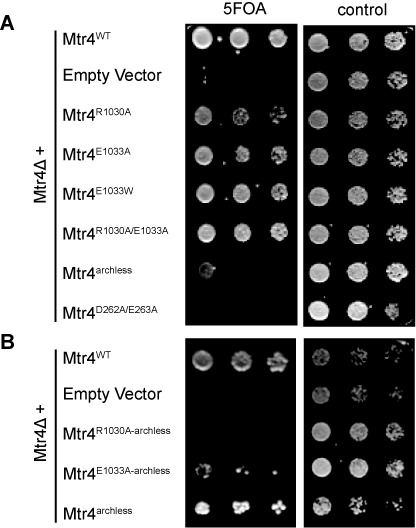
Growth complementation of an Mtr4-knockout strain by ratchet helix mutants. (**A**) Slow growth phenotypes are observed for each of the Mtr4 ratchet helix mutants. The indicated plasmids were introduced into a yeast strain that had the Mtr4^WT^ gene deleted from the chromosome, and that also contained a plasmid encoding Mtr4^WT^ with an URA3 selectable marker. Growth on 5-FOA plates selects for cells that have lost the URA3 plasmid, and thus shows a slow growth phenotype associated with each mutant. Mtr4^archless^ has a more severe slow growth phenotype than any of the ratchet helix mutants. Mtr4^D262A/E263A^ removes residues required for the binding and hydrolysis of ATP and is used as a negative control. (**B**) Double mutants of ratchet helix point mutations combined with an arch deletion. No growth is observed for the Mtr4^R1030A-archless^ mutant and slow growth is observed for the Mtr4^E1033A-archless^ mutant.

After demonstrating that the R1030 and E1033 residues influence Mtr4 helicase activity *in vitro*, we then asked how mutations at ratchet helix positions affect Mtr4 function *in vivo*. Mtr4 mutants, Mtr4^R1030A^, Mtr4^E1033A^, Mtr4^E1033W^ and the double mutant Mtr4^R1030A/E1033A^ were constructed, serially diluted and tested for viability at 20, 30 and 37°C in *S. cerevisiae*. Complementation with plasmid containing Mtr4^WT^ was used as a positive control, whereas Mtr4^archless^ and Mtr4^D262A/E263A^ mutants were used to demonstrate a slow growth phenotype and an active site mutation, respectively. The ratchet helix mutations cause a slow growth phenotype at all temperatures tested when compared to Mtr4^WT^; however, this growth phenotype is less severe than that of Mtr4^archless^ (Figure 4A). This demonstrates that these residues are important for Mtr4 function *in vivo*, although these mutants retain some activity. Furthermore, the Mtr4^D262A/E263A^ double mutation does not compound the growth phenotype observed at single sites, suggesting that defects caused by each ratchet helix mutation disrupt the same mechanistic pathway.

Notably, while each of the ratchet helix mutants confers a slow growth phenotype, none of the mutants are as detrimental as the arch deletion (Mtr4^archless^) on cell viability. Although the fist of the Mtr4 arch domain binds RNA *in vitro* (38), unwinding activity in an arch deletion does not alter unwinding rates (30). To further probe the effect of these mutations, we paired the ratchet helix mutants with an Mtr4^archless^ mutant (Figure 4B). Mutants Mtr4^R1030A-archless^ and Mtr4^E1033A-archless^ were constructed, serially diluted and tested for viability at 30°C. In each case, combination of ratchet helix point mutants with Mtr4^archless^ resulted in a synthetic growth phenotype that was more severe than Mtr4^archless^, with no growth observed for Mtr4^R1030A-archless^. This result suggests that the arch and the ratchet helix are involved in two complementary aspects of Mtr4 function.

### Most Mtr4 mutants have minimal impact on RNA affinity

**Figure 5.**
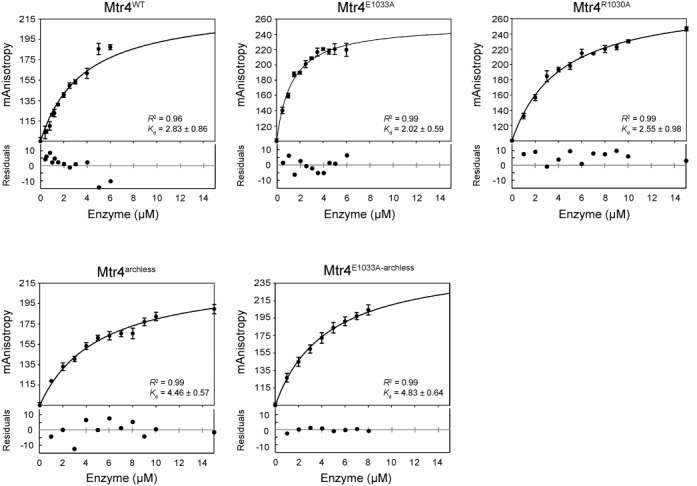
The ratchet helix and the arch mutations have minimal effect on RNA-binding interactions. Fluorescence anisotropy monitored using a fluorescein-labeled poly(A) RNA substrate, plotted as a function of Mtr4 concentration. The correlation coefficient (*R*^2^) and calculated *K*_d_ values (μM) for each curve are inset. Data presented here represent averages from three independent experiments; error bars represent SD. Residual value plots for the curve fits of wild-type and all Mtr4 mutations are shown below their respective curves. All plots passed a residual runs test.

Multiple domains contribute to RNA binding in Mtr4. The Mtr4 RNA-bound crystal structure (38) reveals that the RecA1 and RecA2 domains are important for backbone interactions to the phosphate and sugar of nucleotides. Domain 4 (i.e. ratchet domain) interacts with the bases through hydrogen bonding. Although not observed crystallographically, electrophoretic mobility shift assays (EMSAs) demonstrate that the fist/KOW region of the arch domain binds structured RNAs (38). To determine whether a loss in binding affinity contributed to changes in unwinding activity, we measured poly(A) RNA binding for each of the Mtr4 mutants. *K*_d_*s* were determined by fluorescence anisotropy in the same buffer conditions used for the unwinding assays (Figure 5 and Table 3). Mtr4^WT^ displayed a *K*_d_ of 2.8 μM, and the ratchet helix point mutants Mtr4^R1030A^ and Mtr4^E1033A^ exhibited similar affinities, within the error of the experiment. Archless mutants Mtr4^archless^ and Mtr4^E1033A-archless^ showed a 2-fold decrease in affinity for the poly(A) substrate compared to wild-type. In the case of Mtr4^R1030A-archless^, no binding was detected. Additionally, no binding was detected up to 20 μM by EMSA (Supplementary Figure S5).

**Table 3.** Binding affinities of Mtr4 and mutants on a poly(A) substrate as determined by fluorescence anisotropy^a^

	*K*_d_ (μM)
Mtr4^WT^	2.8 ± 0.9
Mtr4^archless^	4.5 ± 0.6
Mtr4^R1030A^	2.6 ± 1.0
Mtr4^E1033A^	2.0 ± 0.6
Mtr4^R1030A-archless^	> 10
Mtr4^E1033A-archless^	4.8 ± 0.6

Binding affinities > 10 μM were not quantified by anisotropy. Data presented here represent averages from three independent experiments; error bars represent SD.

^a^Methods and equations used to derive kinetic constants are found in the ‘Materials and Methods’ section.

### Deletion of the arch domain abolishes helicase activity when combined with ratchet helix point mutations

The arch domain plays an important but poorly understood role in activation of exosome activity, including Rrp6 (30,42,60). The arch binds structured RNAs but does not bind ssRNA (Figure 5C and (32,38)). Removal of the arch domain does not affect the unwinding rates of Mtr4^WT^ (30). Additionally, as shown in Table 1, the arch is not required for differentiating between a poly(A) and a non(A) substrate. However, when we combine either the Mtr4^R1030A^ or the Mtr4^E1033A^ ratchet helix point mutants with an arch deletion (Mtr4^R1030A-archless^ or Mtr4^E1033A-archless^), unwinding activity is abolished (Table 1 and Figure 2C). The same lack of unwinding activity is observed for TRAMP^E1033A-archless^ (unwinding activity for TRAMP^R1030A-archless^ was undetermined due to poor solubility of the complex). This loss of helicase activity is consistent with the *in vivo* data demonstrating severe growth phenotypes for both Mtr4^R1030A-archless^ and Mtr4^E1033A-archless^ (Figure 4B).

## DISCUSSION

The conservation patterns observed along the ratchet helix of Ski2-like and DEAH/RHA-box helicases suggest that the ratchet helix may play a more extensive functional role in Ski2-like RNA helicases than in other helicases. In order to more clearly define the role of conserved residues along the ratchet helix, we targeted two residues in Mtr4: E1033 and R1030. Mutations at each of these positions result in slow growth phenotypes *in vivo* (Figure 4A). The mutations also alter *in vitro* helicase unwinding activity and ATPase activity, although different effects are observed at each position (Figure 2). While it is uncertain whether the observed slow growth phenotypes are a direct consequence of altering Mtr4 helicase activity, these data clearly underscore the important functional role of the ratchet helix.

R1030 and E1033 both contribute to unwinding of RNA substrates. In the case of R1030, mutation to alanine reduces the unwinding rate (*k*_unw_^max^) by ∼2-fold. For E1033, the effect on unwinding may be related to the size of the side chain. When the glutamate is mutated to a smaller alanine, *k*_unw_^max^ increases, whereas mutation to a larger tryptophan (analogous to Hel308 W599) has the inverse effect (Table 1). Note that this result differs from Brr2 where the analogous mutation to an alanine abolished unwinding (43). The changes in unwinding rate observed in the Mtr4 ratchet helix mutants do not appear to be a function of differences in RNA-binding affinity since no differences in poly(A) substrate binding are observed for Mtr4^R1030A^ or Mtr4^E1033A^ as compared to Mtr4^WT^.

Our studies reveal an important role for R1030 in recognition of a poly(A) tail. This result is consistent with the direct interactions observed between R1030 and an adenine base in the RNA-bound crystal structure of Mtr4 (Figure 1, Supplementary Figure S2). Significantly, both Mtr4 and TRAMP lose the ability to discriminate between poly(A) and non(A) RNA sequences when R1030 is mutated to alanine. R1030 is strictly conserved across Mtr4 sequences. In contrast, Ski2, which is not functionally associated with a poly(A) polymerase, exhibits sequence variability at the equivalent position (a tryptophan occupies that position in *S. cerevisiae* Ski2). The simplest model is that R1030 directly reads the RNA sequence, promoting preferential unwinding of substrates polyadenylated by Trf4 or Trf5. Given the preference for a 3′ tail containing five adenosines (29), it is possible that Mtr4 reads the sequence of multiple nucleotides as the helicase engages the RNA substrate. Thus, additional residues, including other conserved residues along the ratchet helix, may also influence sequence discrimination. One candidate is R1026, which interacts with RNA in the crystal structure (Figure 1, Supplementary Figure S2). We note that R1026 also interacts directly with the RecA2 domain, suggesting a probable structural role that could complicate mutational analysis. Another candidate is E947 (located on the ratchet domain near the ratchet helix), which has been shown to help regulate the length of the poly(A) tail formed by TRAMP (24). A recent low-resolution SAXS reconstruction of a TRAMP complex (61) places Trf4 far away from Mtr4 in a position that makes it difficult to explain the observed interplay between Trf4 polyadenylation and Mtr4 helicase activity, including sensing of a poly(A) tail. Thus, additional structural data are needed to better understand how functional TRAMP complexes assemble, interact with RNA substrates and modulate subunit activity.

**Figure 6.**
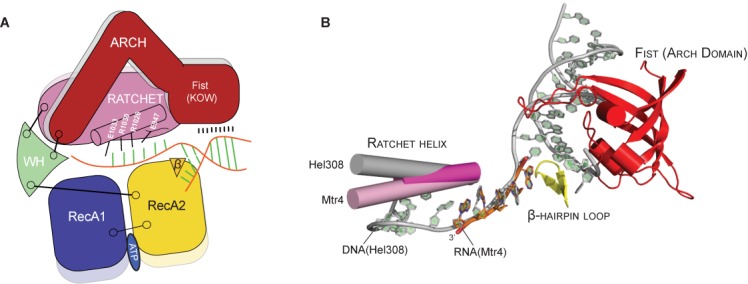
Mtr4 contains an extended RNA-binding interface. (**A**) Cartoon schematic shows observed and predicted interactions between Mtr4 and an unwinding RNA substrate, including direct interactions with the ratchet and arch domains. WH, winged helix domain. (**B**) Alignment of Mtr4 and Hel308 structures reveals conformational differences in the ratchet helix (Hel308, gray; Mtr4 apo, dark pink; Mtr4 RNA-bound, light pink) and predicted interactions with the fist region of the Mtr4 arch domain (red). Structures were aligned using the core helicase domains (RecA1, RecA2, winged helix, and ratchet domains). The DNA substrate observed in the Hel308 structure (gray) contains an unwound duplex region and a single-stranded section that is much longer than the 5nt poly(A) RNA observed in the Mtr4 structure (orange), and highlights the potential physical relationship between the arch domain and the ratchet helix. The figure was rendered with PyMOL (62) using PDB 2P6R (Hel308), 2XGJ (Mtr4–RNA) and 4QU4 (the newly refined Mtr4 apo structure, see ‘Materials and Methods’).

The studies presented here highlight an unexpected relationship between the Mtr4 arch domain and unwinding activity. We previously reported (30) and confirmed in this study (Table 1) that deletion of the arch domain had no observable effect on the unwinding rate. However, combination of the arch deletion with point mutations along the ratchet helix completely abolished helicase activity (Figure 2C and Table 1), regardless of whether the individual ratchet helix mutations caused an increase or decrease in unwinding activity. In the case of the Mtr4^R1030A-archless^ mutant, loss of helicase activity is likely a function of its reduced ability to bind RNA substrates (Table 3 and Supplementary Figure S5). The loss of unwinding activity observed in the Mtr4^E1033A-archless^ mutant, however, is more difficult to explain. While Mtr4^E1033A-archless^ RNA binding is somewhat reduced compared to Mtr4^WT^, the reduction is equivalent to that observed in Mtr4^archless^ (which shows no difference in unwinding rate compared to Mtr4^WT^). Nevertheless, these data establish a relationship between the arch domain and the ratchet helix during unwinding events, and are consistent with the model that Mtr4 contains an extensive substrate binding interface that includes both RecA domains, the ratchet domain, and the arch, and that interactions with each of these domains contribute to unwinding (Figure 6).

Additional studies are needed to clarify the interactions between each domain and with RNA substrates. The dynamic relationship between domains and RNA-binding events is poorly understood. For example, conformational changes are observed between the apo- and RNA-bound forms of Mtr4, including the ratchet helix (40). Even larger differences are observed in the ratchet helix between the Mtr4 RNA-bound structure and the Hel308 DNA-bound structure, with the Mtr4 apo structure occupying an intermediate position (Figure 6B). These conformational differences may arise from the fact that the Mtr4-RNA structure only includes a 5 nucleotide poly(A) sequence while Hel308 is bound to a partially unwound DNA duplex with a single-strand that extends past the ratchet helix and exits through the base of the structure. One potential implication is that recognition of a 3′ poly(A) tail and movement along an RNA substrate involve different conformational states. Engagement of the arch domain with the upstream RNA sequence may also influence RNA interactions along the ratchet helix (Figure 6B). The arch domain potentially helps direct RNA substrates toward the helicase core. One might expect the arch, which is structurally mobile, to adopt alternate conformations as it encounters various structural features in the RNA and/or associated nucleoprotein complexes. Such interactions could affect the path of the unwound RNA strand as it approaches the ratchet helix. Alternatively, conformational changes in the arch could be communicated to the rest of the Mtr4 structure through the winged-helix domain to which all of the other domains are tethered (Figure 6A) (40). Thus, in addition to the 3′ tail of the RNA, the sequence and structural properties of other regions of the RNA substrate may be monitored by Mtr4 and are important considerations when considering the molecular details of Mtr4 function.

## SUPPLEMENTARY DATA

Supplementary Data are available at NAR Online.

SUPPLEMENTARY DATA

## ACCESSION NUMBERS

Accession numbers 4QU4.
