# Supplementary material for: The Mtr4 ratchet helix and arch domain both function to promote RNA unwinding
Source: Nucleic Acids Res. 2014 Nov 20;42(22):13861–72. doi: 10.1093/nar/gku1208 (PMC4267639; doi:10.1093/nar/gku1208)
Supplement: SUPPLEMENTARY DATA [file supp_42_22_13861__index.html]

The Mtr4 ratchet helix and arch domain both function to promote RNA unwinding — The Mtr4 ratchet helix and arch domain both function to promote RNA unwinding — SUPPLEMENTARY DATA 

# The Mtr4 ratchet helix and arch domain both function to promote RNA unwinding

## SUPPLEMENTARY DATA

**Files in this Data Supplement:**

- SUPPLEMENTARY DATA
