# Supplementary material for: The Mtr4 ratchet helix and arch domain both function to promote RNA unwinding
Source: Nucleic Acids Res. 2014 Nov 20;42(22):13861–72. doi: 10.1093/nar/gku1208 (PMC4267639; doi:10.1093/nar/gku1208)
Supplement: SUPPLEMENTARY DATA [file supp_gku1208_nar-01864-r-2014-File008.pdf]

Supplementary Material

The Mtr4 ratchet helix and arch domain both function to promote RNA unwinding

Lacy L Taylor[1], Ryan N Jackson[1], Megi Rexhepaj[1], Alejandra Klauer King[2] Lindsey K Lott[1], Ambro van Hoof[2], and Sean J Johnson[1]

[1] Department of Chemistry and Biochemistry, Utah State University, Logan, UT, 84322 - 0300, USA
[2] Department of Microbiology and Molecular Genetics, University of Texas Health Science Center - Houston
Houston, TX, 77030, USA

Supplementary Figure 1.

A

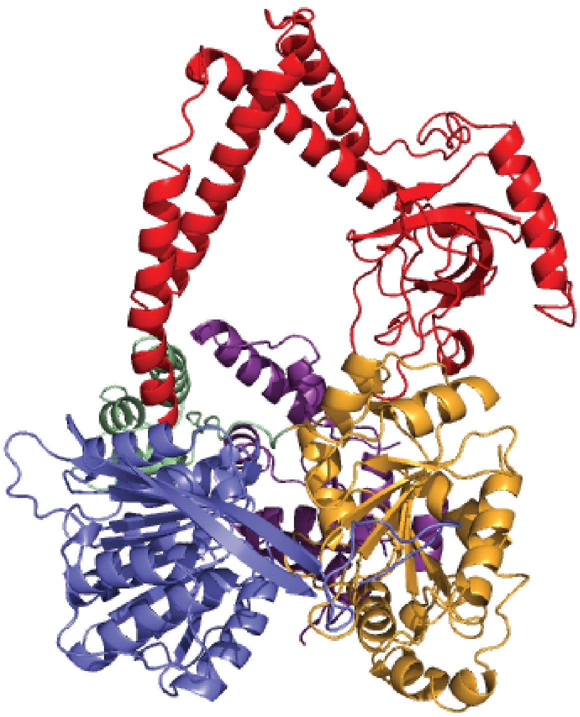

B

1 MDSTDLFDVFEETPVELPTDSNGEKNADTNVGDTPDHTQDKKHGLEEEKEEHEEN

56 NSENKKIKSNKSKTEDKNKKVVVPVLADSFEQEASREVDASKGLTNSETLQVEQD

111 GKVRLSHQVRHQVALPPNYDYTPIAEHKRVNEARTYPFTLDPFQDTAISCIDRGE

166 SVLVSAHTSAGKTVVAEYAIAQSLKNKQRVIYTSPIKALSNQKYRELLAEFGDVG

221 LMTGDITINPDAGCLVMTTEILRSMLYRGSEVMREVAWVIFDEVHYMRDKERGVV

276 WEETIILLPDKVRYVFLSATIPNAMEFAEWICKIHSQPCHIVYTNFRPTPLQHYL

331 FPAHGDGIYLVVDEKSTFREENFQKAMASISNQIGDDPNSTDSRGKKGQTYKGGS

386 AKGDAKGDIYKIVKMIWKKKYNPVIVFSFSKRDCEELALKMSKLDFNSDDEKEAL

441 TKIFNNAIALLPETDRELPQIKHILPLLRRGIGIHHSGLLPILKEVIEILFQEGF

496 LKVLFATETFSIGLNMPAKTVVFTSVRKWDGQQFRWVSGGEYIQMSGRAGRRGLD

551 DRGIVIMMIDEKMEPQVAKGMVKGQADRLDSAFHLGYNMILNLMRVEGISPEFML

606 EHSFFQFQNVISVPVMEKKLAELKKDFDGIEVEDEENVKEYHEIEQAIKGYREDV

661 RQVVTHPANALSFLQPGRLVEISVNGKDNYGWGAVVDFAKRINKRNPSAVYTDHE

716 SYIVNVVVNTMYIDSPVNLLKPFNPTLPEGIRPAEEGEKSICAVIPITLDSIKSI

771 GNLRLYMPKDIRASGQKETVGKSLREVNRRFPDGIPVLDPVKNMKIEDEDFLKLM

826 KKIDVLNTKLSSNPLTNSMRLEELYGKYSRKHDLHEDMKQLKRKISESQAVIQLD

881 DLRRRKRVLRRLGFCTPNDIIELKGRVACEISSGDELLLTELIFNGNFNELKPEQ

936 AAALLSCFAFQERCKEAPRLKPELAEPLKAMREIAAKIAKIMKDSKIEVVEKDYV

991 ESFRHELMEVVYEWCRGATFTQICKMTDVYEGSLIRMFKRLEELVKELVDVANTI

1046 GNSSLKEKMEAVLKLIHRDIVSAGSLYL

Supplementary Figure 1. Overview of newly refined apo-Mtr4 structure (PDB: 4QU4). (A) Cartoon representation of Mtr4 structure, colored by domain. RecA1 (blue), RecA2 (yellow), Winged Helix (green), Arch domain (red), and domain 4 (purple). (B) Mtr4 primary sequence and secondary structure observed in the newly refined model (PDB: 4QU4). A dashed line in the secondary structure plot indicates regions that are not observed in the electron density. Portions of the model that have been significantly changed from the original model (PDB: 3L9O) are indicated in the sequence (red coloring). Underlined residues indicate that complete sidechains have been built where omitted in the original model.

Supplementary Table 1. Comparison of refinement statistics for the newly refined apo-Mtr4 structure (4QU4) compared to the originally submitted coordinates (3L9O).

	**3L9O**	**4QU4**
Refinement		
Resolution (Å)	30-3.4	30-3.4
R_{free}	0.333	0.2991
R_{work}	0.293	0.2475
No. atoms		
Protein	6487	6665
Ligand/ion	20	15
Water	0	0
B-factors		
Protein	157.9	166.1
Ligand/ion	171.2	181.2
RMSD		
Bond lengths (Å)	0.011	0.010
Bond angles (deg)	1.53	1.46
Protein Geometry		
Poor rotamers (%)	10.4	4.4
Ramachandran outliers (%)	8.6	5.9
Ramachandran favored (%)	70.3	83.4

Supplementary Figure 2.

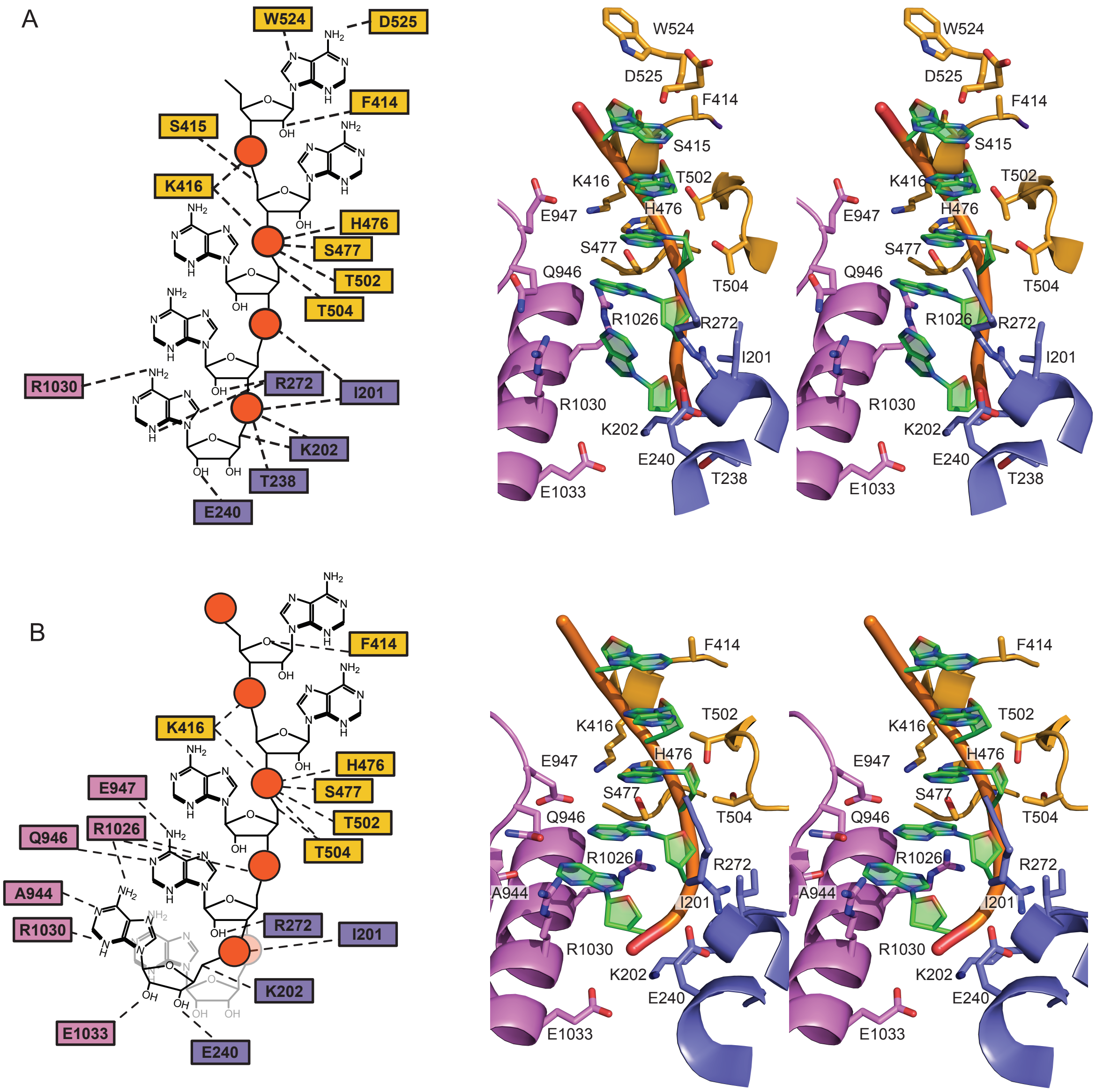

Supplementary Figure 2. Cartoon schematic and structural depiction of the RNA-protein interface of Mtr4 with a 5 nt poly(A) substrate. Two molecules are observed in the asymmetric unit of the Mtr4 structure (PDB: 2XGJ) (1). (A) Chain A. (B) Chain B. Key protein-RNA interactions are highlighted schematically (dashed lines, left) and by stereo view (right). Figure was rendered using ChemDraw (Cambridge Soft) and PyMOL (2).

Supplementary Figure 3.

Ski2 - Like RNA Helicases (Eukarya)

S. cerevisiae **Mtr4** Ratchet Helix

PDB	Species	Helix Turn: 1 2 3 4 5 6
**3L9O/2XGJ**	**S.cer 1021**	EGSLIRMFKRLEELVKELVDVAN
	S.pom 1065	EGSLIRMFRRLEELIRQMVDAAK
	M.bre 888	EGSIIRAMRRLEELLKQMIAAAK
	M.mus 991	EGSIIRCMRRLEELLRQMCQAAK
	D.rer 982	EGSIIRCMRRLEELLRQMCQAAK
	N.cra 1054	EGSLIRLFRRLEELLRQMAEAAR
	H.sap 990	EGSIIRCMRRLEELLRQMCQAAK
	D.mel 1003	EGSIIRCMRRLEELLRQMCQASK
	C.ele 974	EGSIIRTLRRLEEVLREMINAAK
	A.tha 943	EGSIIRSARRLDEFLNQLRAAAE

S. cerevisiae **Ski2** Ratchet Helix

PDB	Species	Helix Turn: 1 2 3 4 5 6 7
**4A4Z**	**S.cer 1236**	EGTVVRVITWLDETCREVKTASIII
	S.pom 1162	EGSIVRTIIRLDEVLRECRGAARVV
	M.bre 946	EGSIVRCITRLDETCRDIRNAAHVV
	M.mus 1192	EGLVVRCIQRLAEMCRSLRGAARLV
	D.rer 1197	EGTIVRCIQRLDEVLKEVRQAARIV
	N.cra 1243	EGTIVRTISRLDETCREVKNAARII
	H.sap 1194	EGLVVRCIQRLAEMCRSLRGAARLV
	D.mel 1144	EGIIVRCIQQLNERVRDVKTAAIRI
	C.ele 1213	EGLIVKCIQRLDEVCKDVRNAGRIV
	A.tha 1284	EGTVVRVIVRLDETCREFKNAAAIM

S. cerevisiae **Brr2** S1 Ratchet Helix

PDB	Species	Helix Turn: 1 2 3 4 5 6
	S.cer 1095	NSDIVFIHQNAGRLLRAMFEICLK
	S.pom 1116	VADMVYVTQSAGRIMRAIFEISLR
	M.bre 1076	MSDMVYITQSAGRLLRAIFEIVLR
	M.mus 1078	MADMVYVTQSAGRLMRAIFEIVLN
	D.rer 1075	MADMVYVTQSAGRLMRAIFEIVLS
	N.cra 1129	MADMVYVTQSAGRILRAIFEITMK
**4BGD**	**H.sap 1078**	MADMVYVTQSAGRLMRAIFEIVLN
	D.mel 1078	MSDMVFITQSAARLMRAIFEIVLT
	C.ele 1071	QADMVFVAQSAGRLFRALFEIVLW
	A.tha 1103	TSDMVYITQSAGRLLRAIFEIVLK

Ski2 - Like DNA Helicase (Archaeal)

S. solfataricus **Hel308** Ratchet Helix

PDB	Species	Helix Turn: 1 2 3 4 5 6 7
**2P6R**	**A.ful 586**	PGDLRRIVETAEWLSNAMNRIAEEVG
2VA8	S.sul 607	SGDLRNMVETMDWLTYSAYHLSRELK
2ZJ8	P.fur 596	PGDIYRIVETAEWLVYSLKEIAKVLG
2Z41	P.hor 590	PGDIYRIVETAEWLVYSLKEIAKTLE
	A.ven 592	PGDLMRVVETAEWLAYALSKIAAFIN
	F.pla 590	PGDLRRIVETAEWLMHSLRRIAEHLS
	M.the 612	PGDLVRIAETAEWLMSALHRISKHMD
	S.hel 656	PGDVYSARDTASWIAGALSRVEKVLG
	T.vol 590	PGDLQARVNNADWISYSLAHLASIFK
	P.aci 622	PGEFYNILENTKWLLYSLKEVAKVLG
	M.kan 639	PGDLYRAKDDAAWIAWGMSRLARAAG

DEAH - Box RNA Helicases (Eukarya)

S. cerevisiae **Prp43** Ratchet Helix

PDB	Species	Helix Turn: 1 2 3 4 5
**2XAU**	**S.cer 610**	YRSLSAADNIRSQLERLMNR
	S.pom 592	HRALISADNVRKQLRRTMER
	M.bre 451	QRSLKRAQDVRKQMVAIMDR
	M.mus 655	YRSLMSADNVRQQLSRIMDR
	D.rer 629	YRSLMSADNVRQQLSRIMDR
	N.cra 623	YRHLSSADNVRAQLKRIMET
	H.sap 655	YRSLMSADNVRQQLSRIMDR
	D.mel 588	FRSLKSADNVRQQLARIMDR
	C.ele 599	YRTMKTADTVRTQLSRVMDK
	A.tha 582	NNRAMKSADNVRQQLVRIMS

H. sapiens **Prp22** Ratchet Helix

PDB	Species	Helix Turn: 1 2 3 4 5
**3I4U**	**H.sap 1069**	ARSLRRAQDIRKQMLGIMDR
	S.pom 1015	ARGMRRAEDVRKQLIRLMDR
	M.bre 1133	AKSMRKVREIRMQLMDIMKS
	M.mus 1093	ARSLRRAQDIRKQNLGIMDR
	D.rer 1059	ARSLRRAQDIRKQMLGIMDR
	N.cra 1018	ARSMRRAKDVRDQIVKIMDR
	S.cer 987	FRHLKRARDVDSQISMIFKK
	D.mel 1090	IRTLKRSQDVRKQLLGIMDR
	C.ele 1044	VRSMKRAQDIRKQLLGIMDR
	A.tha 972	SRSLRRAQDVRKQLLSIMDK

Variable — Conserved

Supplementary Figure 3. Alignment and conservation scoring of the ratchet helix of Ski2-like and DEAH/RHA box helicases. Conservation scores were calculated by ConSurf and presented as described in Figure 1. The PDB ID for structures used to define the helical turn positions on the ratchet helix for each helicase family is indicated to the left of the sequence (bold).

Eukaryotic species: S.cer, *Saccharomyces cerevisiae*; S.pom, *Schizosaccharomyces pombe*; N.cra, *Neurospora crassa*; H.sap, *Homo sapiens*; M.mus, *Mus musculus*; D.rer, *Danio rerio*; D.mel, *Drosophila melanogaster*; C.ele, *Caenorhabditis elegans*; M.bre, *Monosiga brevicollis*; A.tha, *Arabidopsis thaliana*.

Archeal sequences: A.ful, *Archaeoglobus fulgidus*; S.sul, *Sulfolobus solfataricus*; P.fur, *Pyrococcus furiosus*; P.hor, Pyrococcushorikoshi; A.ven, *Archaeoglobus fulgidus*; F. pla, *Ferroglobus placidus*; M.the, *Methanosaeta thermophila* S.hel, Staphylothermus hellenicus; T.vol, *Thermoplasma volcanium* P.aci, *Candidatus Parvarchaeum acidophilus*; M.kan, *Methanopyrus kandleri*.

Supplementary Figure 4.

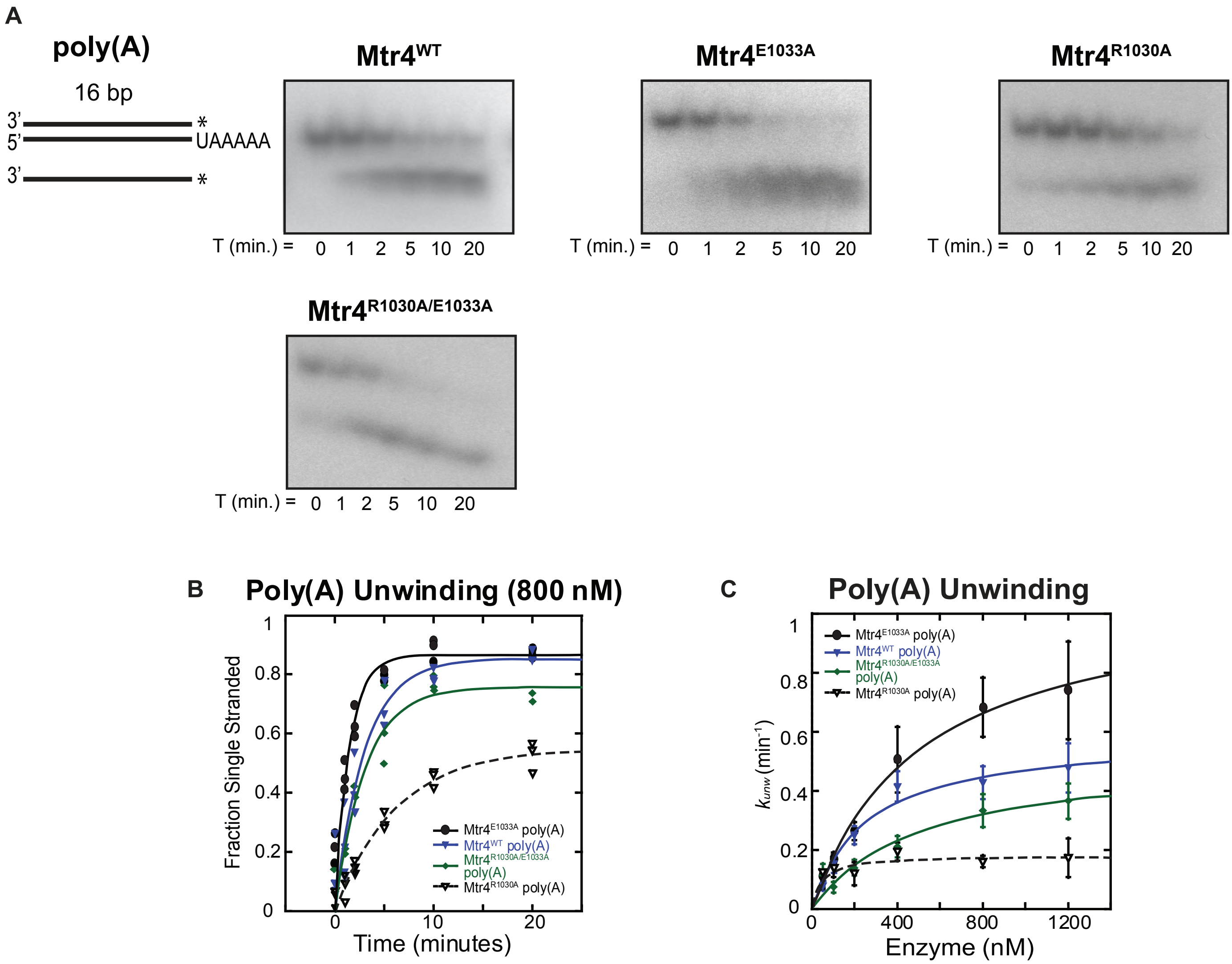

Supplementary Figure 4. Determination of unwinding rate constants for Mtr4^{WT} and ratchet helix point mutants using a poly(A) RNA substrate. (A) Representative native PAGE gels demonstrating displacement of a radiolabeled 16 bp complementary strand by Mtr4 upon addition of ATP as a function of time. All reactions shown correspond to 800 nM enzyme reaction. (B) Representative time course of fraction unwound RNA. The integrated first-order rate law was utilized to generate a best fit curve to the data and the unwinding rate constant (k_{unw}) using KaleidaGraph (Synergy Software). (C) Unwinding rate constants calculated from the time course (as in (B)) plotted against the concentration of Mtr4. The curves represent the best fit to the equation as defined in Jia et al (3). $k_{unw} = k_{max,E}$ [E]/([E] + $K_{1/2,E}$; k_{max} is the maximum unwinding rate, [E] is the enzyme concentration, and $K_{1/2}$ is the functional affinity.

Supplementary Figure 5.

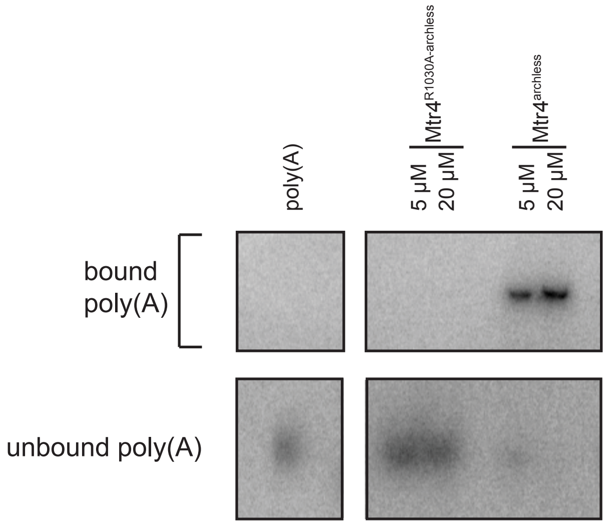

Supplementary Figure 5. Binding to the poly(A) substrate by Mtr4^{archless} is lost when the ratchet helix point mutant is combined (Mtr4$^{R1030A\text{-}archless}$). Using a radiolabeled poly(A) RNA substrate at a concentration of 2 nM, concentrations of protein were varied and incubated for 30 minutes at 30ºC in binding buffer. For Mtr4, a buffer consisting of 40 mM MOPS (pH 6.5), 100 mM NaCl, 0.5 mM $MgCl_2$, 5% glycerol, 0.01% NP-40 substitute, 2 mM DTT, and 1 U/μl of Ribolock (Thermo Fisher) was used (same as unwinding assay buffer). The different concentrations of protein with RNA were then run on a 15% native PAGE gel, exposed to a phosphor screen, and developed by a Storm Phosphorimager. Mtr4$^{R1030A\text{-}archless}$ shows no appreciable binding out to 20 μM protein, whereas Mtr4^{archless} shows almost complete binding at 5 μM protein (consistent with a K_d of 4.46 μM, determined by fluorescence anisotropy).

References:

1. Weir, J.R., Bonneau, F., Hentschel, J. and Conti, E. (2010) Structural analysis reveals the characteristic features of Mtr4, a DExH helicase involved in nuclear RNA processing and surveillance. *Proceedings of the National Academy of Sciences of the United States of America*, **107**, 12139-12144.
2. The PyMOL Molecular Graphics System, Version 1.5.0.4 Schrödinger, LLC.
3. Jia, H., Wang, X., Anderson, J.T. and Jankowsky, E. (2012) RNA unwinding by the Trf4/Air2/Mtr4 polyadenylation (TRAMP) complex. *Proceedings of the National Academy of Science of the United States of America*, **109(19)**: 7292-7.
